# Re-analysis of Single Cell Transcriptome Reveals That the NR3C1-CXCL8-Neutrophil Axis Determines the Severity of COVID-19

**DOI:** 10.3389/fimmu.2020.02145

**Published:** 2020-08-28

**Authors:** Jang Hyun Park, Heung Kyu Lee

**Affiliations:** ^1^Graduate School of Medical Science and Engineering, Korea Advanced Institute of Science and Technology (KAIST), Daejeon, South Korea; ^2^The Center for Epidemic Preparedness, KAIST Institute, KAIST, Daejeon, South Korea

**Keywords:** SARS-CoV-2, COVID-19, neutrophil, CXCL8, glucocorticoid, BAL, scRNA-seq

## Abstract

SARS-CoV-2 infection has recently been declared a pandemic. Some patients showing severe symptoms exhibit drastic inflammation and airway damage. In this study, we re-analyzed published scRNA-seq data of COVID-19 patient bronchoalveolar lavage fluid to further classify and compare immunological features according to the patient’s disease severity. Patients with severe symptoms showed DNA damage and apoptotic features of epithelial cells. Our results suggested that epithelial damage was associated with neutrophil infiltration. Myeloid cells of severe patients showed higher expression of proinflammatory cytokines and chemokines such as CXCL8. As a result, neutrophils were abundant in lungs of patients from the severe group. Furthermore, recruited neutrophils highly expressed genes related to neutrophil extracellular traps. Neutrophil-mediated inflammation was regulated by glucocorticoid receptor expression and activity. Based on these results, we suggest that severe COVID-19 symptoms may be determined by differential expression of glucocorticoid receptors and neutrophils.

## Introduction

In December 2019, SARS-CoV-2 was identified in Wuhan, China. Infection by SARS-CoV-2 causes coronavirus disease 2019 (COVID-19), which is characterized by fever, cough, dyspnea, and myalgia in many cases (1). As of May 26, 2020, more than 5 million cases and 300,000 deaths worldwide have been reported by the World Health Organization (WHO)^1^. Because of its fast spread and severity, WHO declared COVID-19 a pandemic on March 11, 2020 (2).

A recent study suggested that the severity of SARS-CoV-2 infection is between that of SARS-CoV and MERS-CoV (3). The pathology of SARS-CoV-2 involves airway damage, which is caused by viral infection and excessive inflammation. Especially in severe cases, acute respiratory distress syndrome (ARDS) is accompanied by difficulty in breathing and low blood oxygen levels. ARDS accounts for 70% of deaths in fatal COVID-19 cases (4). Although proper immune responses are required to eliminate viruses, excessive inflammation can cause severe symptoms in patients.

Proinflammatory cytokines such as IL-1β, TNF, and IFNγ are upregulated in infected patients, when compared to healthy controls (1). Currently, a single cell RNA sequencing (scRNA-seq) study suggested that proinflammatory monocyte-derived macrophages correlate with severe symptoms, and clonally expanded CD8 T cells correlate with moderate symptoms in patients (5). This study suggested that proper regulation among immune cells is important for the moderate pathogenesis and clearance of viral particles. According to the original study, excess macrophage inflammation and clonal expansion of CD8 T cells are highly related to COVID-19. However, the previously published analysis of other cell types, including neutrophils and B cells, was limited. Therefore, we re-analyzed published scRNA-seq data to further investigate the difference between mild and severe COVID-19 patients. Although recruitment of neutrophils in severe COVID-19 patients with severe ARDS-like symptoms was already known, detailed mechanisms of how neutrophils are regulated and contribute to severe COVID-19 symptoms remain unclear (1). Therefore, we focused on the mechanism of how myeloid cells and neutrophils damage the airway and induce severe symptoms.

## Results

### Epithelial Cells From Severe COVID-19 Patients Were More Damaged and Apoptotic Than Those From Mild Patients

Because the severity of COVID-19 is known to be affected by an imbalance of immune responses, we analyzed immune cell types in bronchoalveolar lavage fluid (BALF) from healthy controls and patients with mild and severe symptoms (Supplementary Table S1). For analysis, we downloaded scRNA-seq data (5). Three mild groups and six severe/critical groups were divided by diagnostic criteria.

**FIGURE 1:**
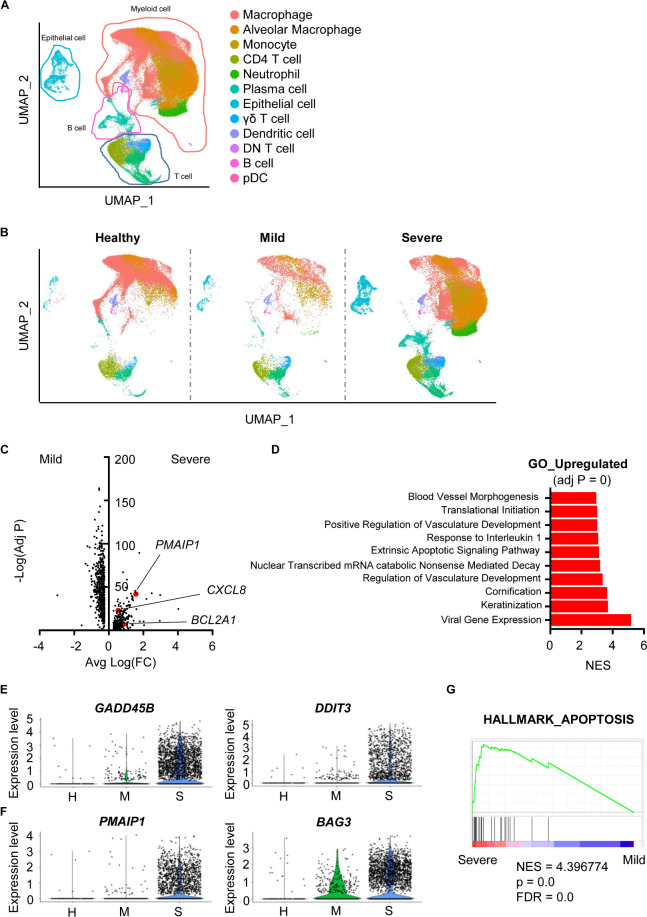
Damaged features of epithelial cells from severe COVID-19 patients. **(A)** The Uniform Manifold Approximation and Projection (UMAP) presentation of 12 major cell clusters in bronchoalveolar lavage fluid scRNA-seq data. **(B)** The UMAP presentation of cell clusters was divided into healthy, mild, and severe groups. **(C)** A volcano plot of differentially expressed genes of epithelial cells between the mild and severe groups. The *x*-axis denotes the average log-scaled fold change of gene expression. The *y*-axis denotes the log-scaled Bonferroni-adjusted p value. **(D)** Upregulated pathways of the severe COVID-19 group compared to the mild group. The adjusted p values of all presented pathways are zero. Pathways were compared using the normalized enrichment score (NES). **(E)** A violin plot of the expression of *GADD45B and DDIT3* of epithelial cells from the healthy (H), mild (M) and severe (S) groups. **(F)** A violin plot of *PMAIP3* and *BAG3* expression in epithelial cells from the H, M, and S groups. **(G)** Gene Set Enrichment Analysis of the sets compared between severe and mild groups with the HALLMARK_APOPTOSIS gene set.

To analyze scRNA-seq data, we referred to the Seurat platform workflow and the original article (5, 6). Using FeaturePlot data and differentially expressed marker genes among clusters, we identified 12 cell lineages. Macrophages (*LYZ*, *CD68*), alveolar macrophages (*CD68*, *SIGLEC1*), monocytes (*CD14*), neutrophils (*FCGR3B*), CD4 T cells (*CD3E*, *TRAC*, *CD4*), CD8 T cells (*CD3E*, *TRAC*, *CD8A*), plasma cells (*IGHG4*, *MZB1*), B cells (*MS4A1*, *CD19*), γδ T cells (*TRDC*), double-negative T cells (*CD3E*, *TRAC*), plasmacytoid dendritic cells (*LILRA4*, *IRF7*), dendritic cells (*CLEC9A*, *CD1A*) and epithelial cells (*KRT18*, *KRT19*) were identified (Figure 1A, Supplementary Table S2 and Supplementary Figure S1). The overall pattern of clusters differed among groups (Figure 1B).

Airway damage is one of the key characteristics of COVID-19. Furthermore, severe patients show ARDS symptoms (4). Thus, we hypothesized that accumulated damage by COVID-19 occurs at the cellular level in the airway constructing cells. A total of 1,011 genes were significantly and differentially expressed between epithelial cells from mild and severe patients (Figure 1C). Cells from severe cases showed higher gene expression of vasculature-related features. Severe cases also showed an extrinsic apoptosis pathway (Figure 1D). DNA damage-associated genes (*GADD45B*, *DDIT3*) and apoptosis-associated genes (*PMAIP1*, *BAG3*) were upregulated in severe patients, while mild patients showed no significant increase compared to healthy controls (Figures 1E,F). Differentially expressed genes (DEGs) from severe patients were highly enriched in the apoptosis-related gene set (Figure 1G). Overall, although SARS-CoV-2 infection did not induce serious epithelial damage, severe patients showed increased DNA damage and apoptotic features of epithelial cells at the transcriptional level.

### Severe Patients Showed a Myeloid Cell-Mediated Cytokine Storm

**FIGURE 2:**
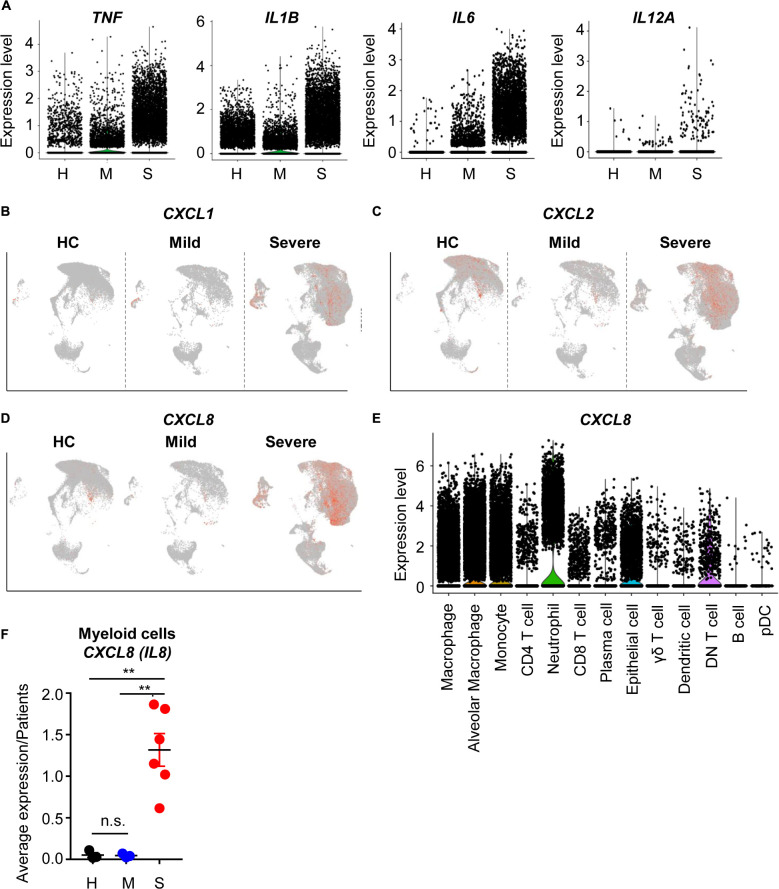
The inflammatory landscape of myeloid cells from severe COVID-19 patients. **(A)** A violin plot of *TNF*, *IL1B*, *IL6*, and *IL12A* expression. **(B–D)** A feature plot of *CXCL1* expression **(B)**, *CXCL2* **(C)**, and *CXCL8* **(D)**. **(E)** A violin plot of *CXCL8* expression in each cluster. **(F)** The average expression level of *CXCL8* from myeloid cells for patients by groups. The data were analyzed using Student’s *t*-test. **p < 0.01. Error bars denote the mean ± SEM.

To determine whether COVID-19-mediated epithelial damage was associated with severe inflammation, we first analyzed the expression patterns of proinflammatory cytokines. Proinflammatory cytokines (*TNF*, *IL1B*, *IL6*, *IL12A*) were mainly produced by myeloid cells including macrophages, alveolar macrophages, and monocytes (Supplementary Figure S2A). As expected, these cytokines were more highly expressed by myeloid cells in severe patients than in other patients (Figure 2A). Thus, we confirmed that cytokine storm-mediated severe inflammation was a signature of severe COVID-19 patients. Not only cytokines, but chemokine expression was also upregulated in severe patients; specifically, recruitment of neutrophils by chemokine was upregulated (Figures 2B–D). Neutrophil-attracting *CXCL8* was mainly produced by epithelial and myeloid cells (Figures 2D,E). Furthermore, *CXCL8* expression was highly enriched in severe cases (Figure 2F); however, mild patients showed no increase of *CXCL8* compared to healthy controls. These results indicated that neutrophil chemotaxis was induced by severe COVID-19, although infection alone did not induce neutrophil chemotaxis. However, *IL17A* expression, which is related to neutrophil-mediated immune responses, did not change in severe cases (Supplementary Figure S2B) (7). Upon further analysis of DEGs in myeloid cells, chemotaxis related genes were also found to be upregulated in eosinophils and monocytes. In addition, negative regulation of the viral process pathway was upregulated in severe patients (Supplementary Figure S2C). These results suggested that myeloid cells, which were directly infected with SARS-CoV-2, were trying to suppress replication. However, their antigen presenting capacity was downregulated in severe cases (Supplementary Figures S2D,E). Although, myeloid cells of severe patients produced more cytokines and chemokines because of infection, their ability to activate T cells, a critical part of the antiviral response, was dysregulated at the transcriptional level. Overall, these results are consistent with previous studies emphasizing the role of T cells in eliminating SARS-CoV-2 (5).

### Recruitment and Degranulation of Neutrophils Was Induced in Severe COVID-19 Patients

Previous results implied that neutrophil chemotaxis is upregulated in severe COVID-19 patients. Although neutrophils are mainly related to fungal and bacterial infections, previous studies suggested that neutrophils are also associated with coronavirus infection. NETosis by neutrophils has been observed in COVID-19 patients (8), and patients needing intensive care unit (ICU) care had higher neutrophil numbers in their blood than non-ICU patients (1). Thus, the relationship between COVID-19 and neutrophils appeared to be important.

**FIGURE 3:**
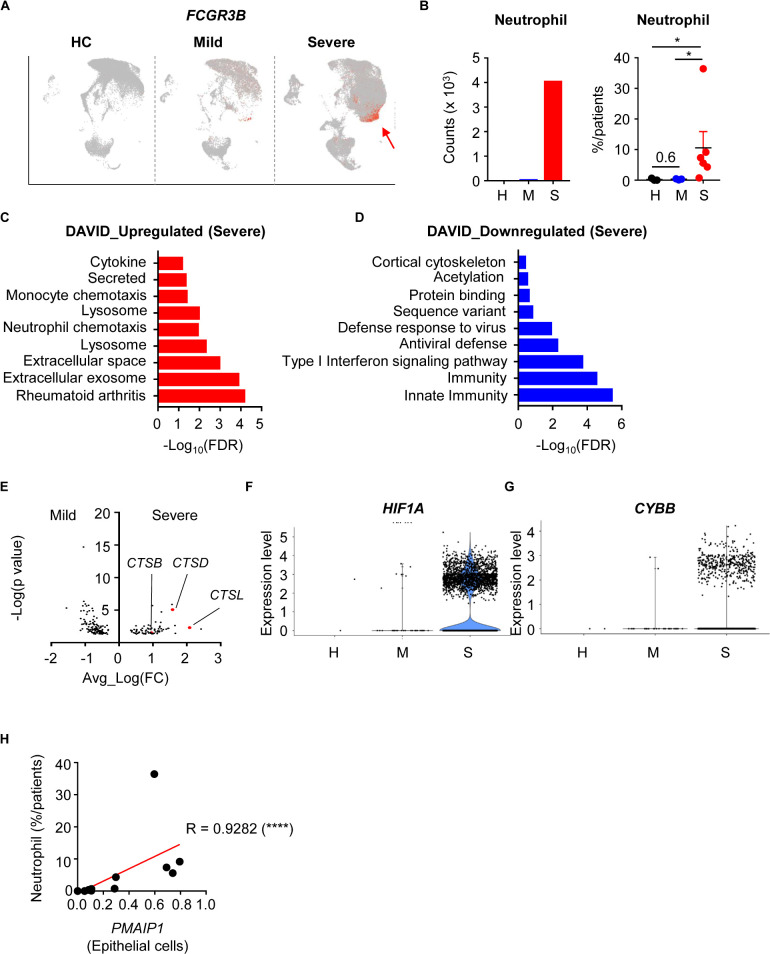
Infiltration and activation of neutrophils in severe COVID-19 patients. **(A)** A feature plot of the expression of *FCGR3B* to identify neutrophil clusters. **(B)** Neutrophil counts of the integrated data (left) and the percentage of neutrophils per patient (right) were compared among the patient groups. Statistical analysis was tested using Student’s *t-*test. **(C,D)** The top 10 upregulated **(C)** and downregulated **(D)** pathways in neutrophils of severe patients compared to mild patients. **(E)** Differentially expressed genes in neutrophils. **(F,G)** Violin plots of *HIF1A* expression **(F)** and *CYBB* expression **(G)** in neutrophils by group. **(H)** Correlation of *PMAIP1* expression between epithelial cells and the percentage of neutrophils in the lungs of patients. The correlations were tested using the one-tailed Spearman’s test. *p < 0.05; ****p < -0.0001. Error bars denote the mean ± SEM.

During SARS-CoV-2 infection, patients showed slightly increased neutrophil numbers. However, severe patients showed dramatically more neutrophils in their BALF (Figures 3A,B). To determine the functional differences between mild and severe patients, we compared the DEGs of neutrophils. When we analyzed neutrophil pathways using Database for Annotation, Visualization and Integrated Discovery (DAVID), pathways involving extracellular events, lysosomes, and secretion were upregulated in the severe group (9, 10) (Figure 3C). However, pathways related to immune responses and antiviral defense were downregulated (Figure 3D). These results implied that neutrophils of severe patients were secreting molecules into the extracellular space while antiviral functions were impaired.

Neutrophils are known to secrete neutrophil extracellular traps (NETs) including DNA and extracellular fibers (11). Although NETs can protect the host from pathogens, they can also damage organs. Progranulin proteases, reactive oxygen species (ROS), and granule proteins are needed for NETosis. When we compared DEGs in mild and severe disease groups, cathepsin family protein genes (*CTSB*, *CSTD*, *CTSL*) were upregulated in the severe group (Figure 3E). *HIF1A* and *CYBB* expression, which are related to ROS, were also upregulated in the severe group (Figures 3F,G). As a result, genes for hydrolase activity required for NETs were enriched in the severe group (Supplementary Figure S3A), as well as genes that are upregulated in infection with bacteria such as *F. Tularensis* (Supplementary Figures S3B,C). However, small molecule metabolism-related genes were downregulated in the severe group (Supplementary Figure S3D). As a result, neutrophil infiltration was positively correlated with lung epithelial damage markers (Figure 3H). Taken together, these results showed that neutrophils in the severe disease group were more recruited and activated to make NETs, which related to epithelial damage. However, their metabolism and antiviral responses were dysregulated at the transcriptional level.

### Glucocorticoid Receptor Expression Was Negatively Related With Neutrophilic Inflammation

**FIGURE 4:**
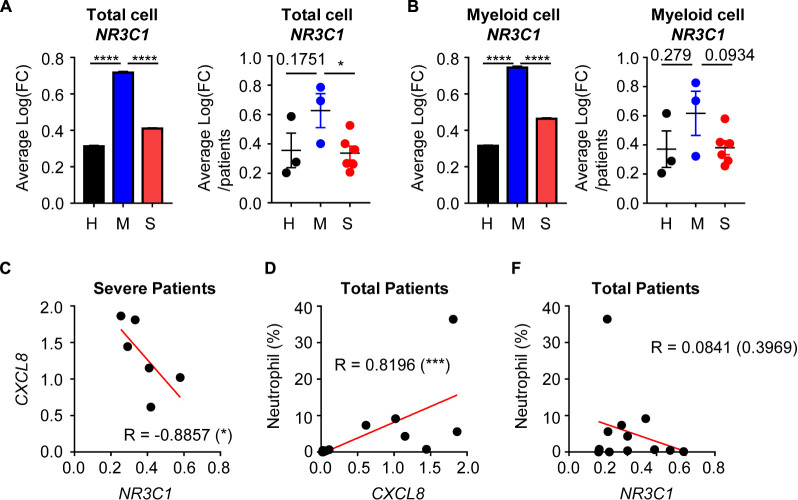
The relationship between glucocorticoid receptors and the CXCL8-neutrophil axis. **(A)** Expression of *NR3C1* of integrated total cells (left) and average expression per patient by group (right). **(B)** The expression of *NR3C1* of integrated myeloid cells (left) and the average expression per patient by group (right). Student’s *t*-test was used for statistical analyses. **(C)** The correlation between *NR3C1* and *CXCL8* in myeloid cells of severe patients. **(D)** The correlation between *CXCL8* in myeloid cells and the percentage of neutrophils. **(E)** The correlation between *NR3C1* in myeloid cells and the percentage of neutrophils. One-tailed Spearman’s test was used for the correlation analyses. *p < 0.05; ***p < 0.001; ****p < 0.0001. Error bars denote the mean ± SEM.

Although evidence is incomplete, previous studies have suggested that glucocorticoid therapy could be effective in COVID-19 treatment (12). Because glucocorticoids have anti-inflammatory activity, they could reduce the cytokine storm and excessive inflammation. We therefore analyzed the possible relationship between glucocorticoid and neutrophil-mediated severity of COVID-19. There was average expression of the glucocorticoid receptor (GR) gene, *NR3C1*, in total cells. In the mild disease group, *NR3C1* expression was increased over that in healthy controls, but was downregulated in the severe disease group (Figure 4A). According to previous results, myeloid cell-derived neutrophil chemotaxis is related to COVID-19 disease severity. Thus, we determined *NR3C1* expression in myeloid cells and found that *NR3C1* expression was upregulated in the mild disease group and downregulated in the severe disease group (Figure 4B). GR activation is known to suppress CXCL8 production, and we found that *NR3C1* expression was negatively correlated with *CXCL8* expression (Figure 4C). As expected, *CXCL8* expression was positively correlated with the percentage of neutrophils (Figure 4D). As a result, *NR3C1* expression was negatively correlated with the percentage of neutrophils (Figure 4E). Further, according to the Gene Set Enrichment Analysis (GSEA) data, upregulated genes of myeloid cells from the severe disease group were negatively enriched in the gene set of GR activity (Supplementary Figure S4A). Taken together, these results showed that GR activity and expression were negatively correlated with neutrophil-mediated severe inflammatory features induced by COVID-19.

Obesity is currently known to be a risk factor for COVID-19 (13). Because obesity causes downregulation of GRs in myeloid cells, we analyzed a public database to find possible links between our data and physiological conditions. We downloaded the average body mass index (BMI) of countries in 2014 and the number of detected SARS-CoV-2 cases and deaths. The average BMI was positively correlated with detection cases, death cases, and mortality (Supplementary Figures S4B–D). These results confirmed that obesity was associated with COVID-19. Furthermore, metabolic disorder-mediated regulation of GR was possibly related to severe symptoms of COVID-19.

## Discussion

In this study, we compared the immunological features of severe and mild COVID-19 patients using scRNA-seq analyses. Previous studies have suggested that the severity of COVID-19 is caused by an imbalance of immune responses. Because of severe inflammation and a cytokine storm, ARDS symptoms were observed in severe patients. This dysregulated inflammation could induce airway damage (4). In parallel, we observed epithelial cell damage as determined by DNA damage and apoptosis at the transcriptional level. We hypothesized that this airway damage was caused by inflammation.

A previous study showed that cytokine secretion from macrophages is a critical determinant of COVID-19 severity (5). Our results also supported the possibility that myeloid cell-derived proinflammatory cytokines such as IL-1β, IL-6, and TNF are produced in larger quantities in the severe disease group. Furthermore, we observed that neutrophil-recruiting chemokines such as CXCL8 from myeloid cells were highly increased as the severity increased. Proinflammatory cytokines such as TNF can increase the expression of CXCL8 and neutrophil chemotaxis (14). Similarly, the number of neutrophils in blood is higher in ICU patients than in non-ICU patients (1). However, we observed that antigen presentation pathways were downregulated in macrophages in the severe disease group. A previous study suggested that clonal T cell expansion was important to clear SARS-CoV-2 (5). Thus, macrophage-mediated antigen presentation and activation could affect the severity of COVID-19.

Consistent with the higher expression of CXCL8, our results showed increased recruitment of neutrophils into the lung. Neutrophils of the severe disease group showed high expression of secretion-related molecules, lysosome-associated molecules, and NETosis features. As suggested by a previous study, sera from COVID-19 patients showed elevated NETs compared to healthy controls (8). Thus, NETs from neutrophils might be involved in COVID-19 disease progression. However, whether neutrophils are related to the severity of the disease and the underlying mechanism, remain unclear. Our data may indicate that recruitment of neutrophils and NETosis could contribute to severity of the disease symptoms through epithelial damage. Although neutrophil genes related to NETosis were increased in the severe group, their antiviral defense system was abnormal. Our findings should be confirmed at the protein level. Furthermore, the detailed mechanism regulating neutrophils should be further studied. For example, although IL-17A is an important cytokine in neutrophil-mediated immune responses (7), our results showed that IL-17A expression was not detectable.

Because excessive inflammation is a feature of severe COVID-19, clinicians have proposed the use of glucocorticoids as a therapy (12). Glucocorticoids such as dexamethasone are known to be anti-inflammatory agents (15). Glucocorticoid treatment reduces TNF-mediated CXCL8 production, according to a previous study (16). Despite the importance of glucocorticoids to deal with inflammation, the role and mechanism of glucocorticoids in COVID-19 treatment is unclear. Thus, we analyzed the relationship between neutrophil recruitment and GR expression. Surprisingly, mild patients showed increased GR expression while severe patients showed reduced GR expression, which was similar to the levels in healthy controls. Our results showed that GR expression was negatively correlated with CXCL8 expression and neutrophil recruitment, suggesting that GR expression and activation regulated neutrophil chemotaxis. Thus, using glucocorticoids to treat severe patients might be effective. Because glucocorticoids are anti-inflammatory agents, glucocorticoid treatment during early disease time points might be harmful. However, late treatment could not reverse airway damage. Thus, the protocol schedule for glucocorticoid therapy will be important. IL-8 blockade might be more effective than glucocorticoid treatment, which may be too broad in its targeting, and the efficacy of anti-IL-8 on SARS-CoV-2 is currently being tested in an ongoing phase 2 clinical trial (NCT04347226). Although the finding that glucocorticoids may be related with neutrophilic inflammation is novel, it should be confirmed at the protein level in future studies.

Obesity has been recently shown to be a risk factor for COVID-19 (17). The proposed mechanisms involve respiratory dysfunction, comorbidities, and metabolic risks (13). Obesity is also a risk factor for influenza infection (18), and correlates with metabolic and respiratory dysfunction, as well as immune response dysfunction. Obesity can cause abnormalities of CD8 T cells, regulatory T cells, and lymphatic vessels (19–21). Thus, an abnormal immune system could be a mechanism of obesity-mediated COVID-19 disease severity. A previous study reported obesity-induced downregulation of GR in Kupffer cells. Reduction of GRs causes liver inflammation (22). Because our results showed that GR expression negatively correlated with neutrophil recruitment, the reason that GR expression was differentially regulated is important. According to previous results and the results of our study, metabolic stress could be a cause of reduced GR expression. Thus, we hypothesize that metabolic disorders such as obesity regulate GR expression and cause the neutrophil-mediated severe symptoms of COVID-19. This hypothesis should be confirmed by further study.

### Data and Code Availability

The scRNA-seq data used in this study is publicly available in GEO (GSE145926).

### Experimental Model and Subject Details

Raw data of the scRNA-seq of patients were obtained from the publicly shared database, GEO (GSE145926). Briefly, patients were divided by symptoms as healthy, mild, or severe. The median age of patients was 57 years. Six males and three females were included (5) (Supplementary Table S1).

### Single Cell RNA Sequencing

Raw data of scRNA-seq from the BALF of patients were obtained from the GEO database (GSE145926). The following analysis was referred to the Seurat R package, version 3.1.5. We discarded cells expressing <200 genes. Furthermore, we discarded cells expressing >20–25% mitochondrial genes. Then, gene expression was normalized. Variable genes from cells were identified by the FindVariableFeatures function using the vst method. Datasets for each group were integrated using the FindIntegrationanchors and IntegrateData functions. Samples were scaled and principal component analysis was conducted using 100 npcs. The cells were then clustered using the FindClusters function with 1.2 resolution. Finally, the data were visualized by UMAP using 1:50 dimensions.

### Cell Type Annotation by Marker Genes

To identify the cell type of clusters, differentially expressed genes of each cluster were gathered using the FindMarkers function. Differentially expressed genes were filtered using the Bonferroni-adjusted *p* value (<0.05). Among 31 clusters, 12 known cell types were annotated (Supplementary Table S2).

### Visualization of Gene Expression

To compare interested gene expression in each cell type or group, VlnPlot and FeaturePlot were used. Some results were shown by volcano plots. To compare gene expression in each cell, all gene expression data of each cell were collected and compared. However, to compare gene expression in patients, the mean expression of interested genes of each patient was calculated and compared.

### Pathway Analysis

For pathway analysis, we used DAVID 6.8^2^. Differentially expressed genes filtered by adjusted p value of each group were uploaded. We used results from Gene Ontology (GO) enrichment analysis. The top 10 enriched pathways were selected by enrichment score and displayed. To compare pathway enrichment specifically, we used Gene Set Enrichment Analysis (GSEA). Differentially expressed genes were annotated to the reference gene set based on the ENSEMBL gene from MSigDB 7.1.

### Correlation Analysis

To test correlations between different genes, we used Spearman’s correlation tests. To analyze correlations between the body mass index (BMI) and the detected cases and death rates of COVID-19 among countries, we downloaded data of the average BMI of populations during 2014 from the WHO. To obtain information about statistics of global COVID-19, we downloaded data from “Our World in Data”^3^ Pearson’s correlation test was used to test correlations between BMI and detected cases and death rates of COVID-19.

### Statistical Analysis

Data are expressed as the mean ± SEM. Differences between experimental groups were analyzed using the unpaired, two-tailed Student’s *t*-tests. Correlations between genes were analyzed by one-tailed Spearman’s correlation tests. However, Pearson’s correlation test was used to test relationships between the BMI and detected cases and death rates of COVID-19. Prism software (GraphPad, San Diego, CA, United States) or R software (R Foundation for Statistical Computing, Vienna, Austria) were used to analyze statistics. Differences were considered statistically significant at *p* < 0.05.

## Data Availability Statement

The raw data supporting the conclusions of this article will be made available by the authors, without undue reservation.

## Author Contributions

JP and HL designed, conducted, and conceived the study, analyzed the data, and wrote the manuscript. Both authors contributed to the article and approved the submitted version.

## Conflict of Interest

The authors declare that the research was conducted in the absence of any commercial or financial relationships that could be construed as a potential conflict of interest.

## Footnotes

1 https://covid19.who.int/

2 https://david.ncifcrf.gov/

3 https://ourworldindata.org/coronavirus
