# Supplementary material for: Re-analysis of Single Cell Transcriptome Reveals That the NR3C1-CXCL8-Neutrophil Axis Determines the Severity of COVID-19
Source: Front Immunol. 2020 Aug 28;11:2145. doi: 10.3389/fimmu.2020.02145 (PMC7485000; doi:10.3389/fimmu.2020.02145)
Supplement: Supplementary file 1 [file Data_Sheet_1.PDF]

# Table S1. Information for patients enrolled in this study

| | M1 | M2 | M3 | S1 | S2 | S3 | S4 | S5 | S6 |
|---|---|---|---|---|---|---|---|---|---|
| **Severity** | Mild | Mild | Mild | Severe | Critical | Critical | Critical | Critical | Critical |
| **Hospital** | Shenzhen Third People's hosptial | | | | | | | | |
| **Criteria** | Diagnosis and Treatment Protocol of COVID-19 (7th Tentative Version)<br>National Health Commission of China (2020-03-03) | | | | | | | | |
| | Fevere, Respiratory symptoms, pneumonia (CT), Moderate infection with bilateral pneumonia | | | Respiratory distress (rate ≥ 30 time/min), fingertip oxygen saturation ≤ 93% (resting state), $PaO_2/FiO_2$ ≤ 300 mm Hg, Obvious progression of lesions by pulmanory imaging > 50% (24-48 h) | Respiratory failure and artificial airway, Shock, Combined failure of other organs | | | | |
| **Age** | 36 | 37 | 35 | 62 | 66 | 63 | 65 | 57 | 46 |
| **Onset date** | 2020-01-09 | 2020-01-11 | 2020-01-09 | 2020-01-11 | 2020-01-03 | 2020-01-08 | 2020-01-04 | 2020-01-21 | 2020-01-21 |
| **Hospitalization date** | 2020-01-16 | 2020-01-16 | 2020-01-15 | 2020-01-18 | 2020-01-11 | 2020-01-15 | 2020-01-20 | 2020-01-23 | 2020-01-22 |
| **BALF sampling date** | 2020-01-20 | 2020-01-20 | 2020-01-22 | 2020-01-22 | 2020-01-21 | 2020-01-22 | 2020-01-29 | 2020-01-29 | 2020-02-02 |
| **Outcome** | Cured | Cured | Cured | Cured | Death | Death | Cured | Cured | Cured |
| **Outcome date** | 2020-01-27 | 2020-01-23 | 2020-02-03 | 2020-02-27 | 2020-02-16 | 2020-02-16 | 2020-03-09 | 2020-03-07 | 2020-03-08 |
| **Chronic basal disease** | None | None | None | None | Hypertension | Sleep apnea | Diabetes | None | None |
| **Gender** | Male | Female | Male | Male | Male | Male | Female | Female | Male |

# Table S2. Markers for identifying the cell lineages of clusters

| | Identity | Markers |
|---|---|---|
| **0** | Macrophage | LYZ, CD68 |
| **1** | Alveolar Macrophage | CD68, SIGLEC1 |
| **2** | Monocyte | CD14 |
| **3** | Macrophage | LYZ, CD68 |
| **4** | Alveolar Macrophage | CD68, SIGLEC1 |
| **5** | CD4 T cell | CD3E, TRAC, CD4 |
| **6** | Neutrophil | FCGR3 |
| **7** | Macrophage | LYZ, CD68 |
| **8** | Monocyte | CD14 |
| **9** | Alveolar Macrophage | CD68, SIGLEC1 |
| **10** | Macrophage | LYZ, CD68 |
| **11** | CD8 T cell | CD3E, TRAC, CD8A |
| **12** | Macrophage | CD68 |
| **13** | Macrophage | LYZ, CD68 |
| **14** | Macrophage | CD68 |
| **15** | Plasma cell | IGHG4, MZB1 |
| **16** | Alveolar Macrophage | CD68, SIGLEC1 |
| **17** | Macrophage | CD68 |
| **18** | Epithelial cell | KRT18, KRT19 |
| **19** | CD8 T cell | CD3E, TRAC, CD8A |
| **20** | Epithelial cell | KRT18, KRT8 |
| **21** | gd T cell | TRDC |
| **22** | Macrophage | LYZ, CD68 |
| **23** | Macrophage | CD68 |
| **24** | Dendritic cell | CLEC9A, CD1A |
| **25** | Plasma cell | IGHG4, MZB1 |
| **26** | DN T cell | CD3E, TRAC |
| **27** | Macrophage | LYZ, CD68 |
| **28** | B cell | MS4A1, CD19 |
| **29** | pDC | LILRA4, IRF7 |
| **30** | Macrophage | CD68 |

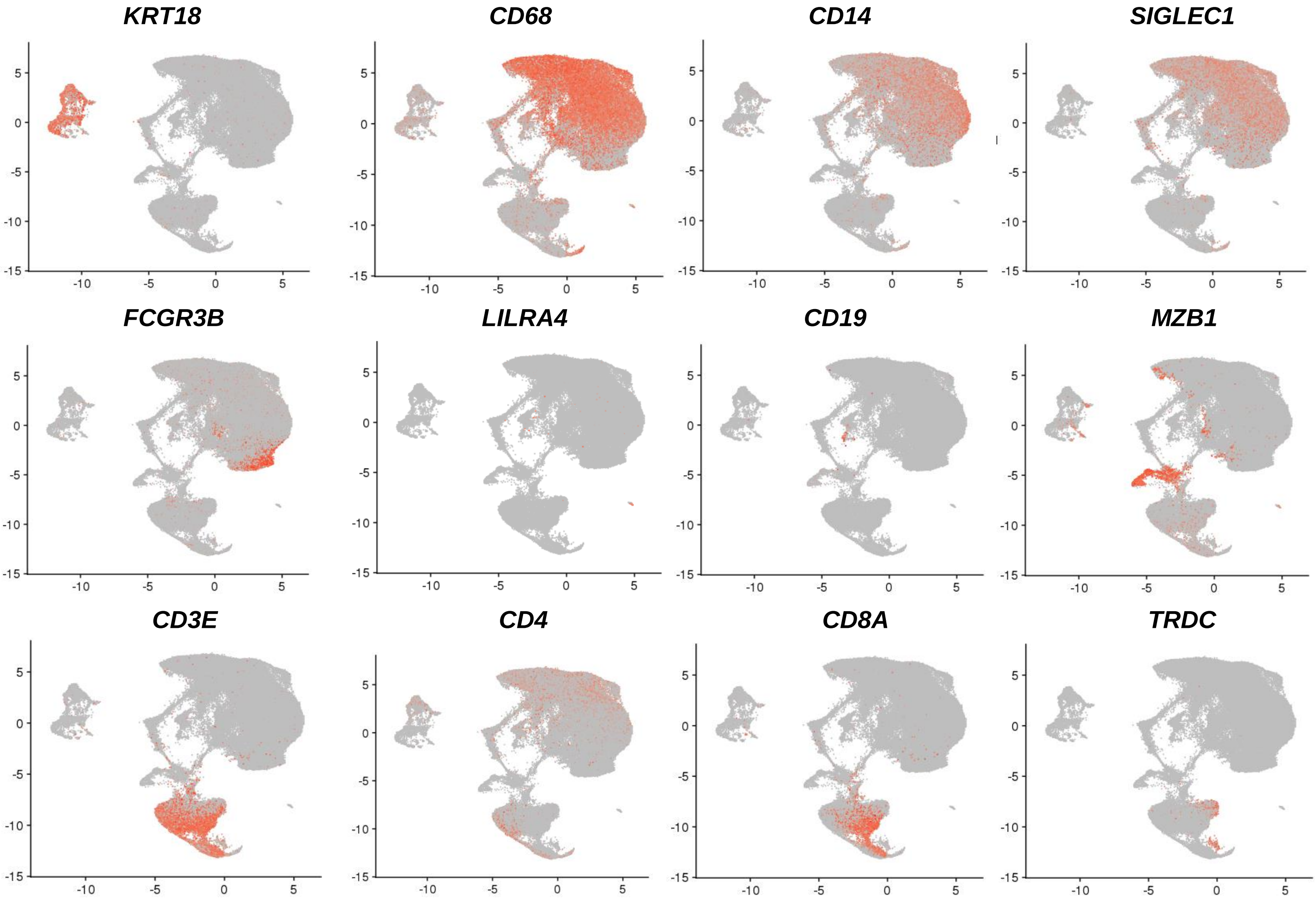


**Figure S1 Identification of cell lineage clusters, related to Figure 1.** The feature plot of representative markers.

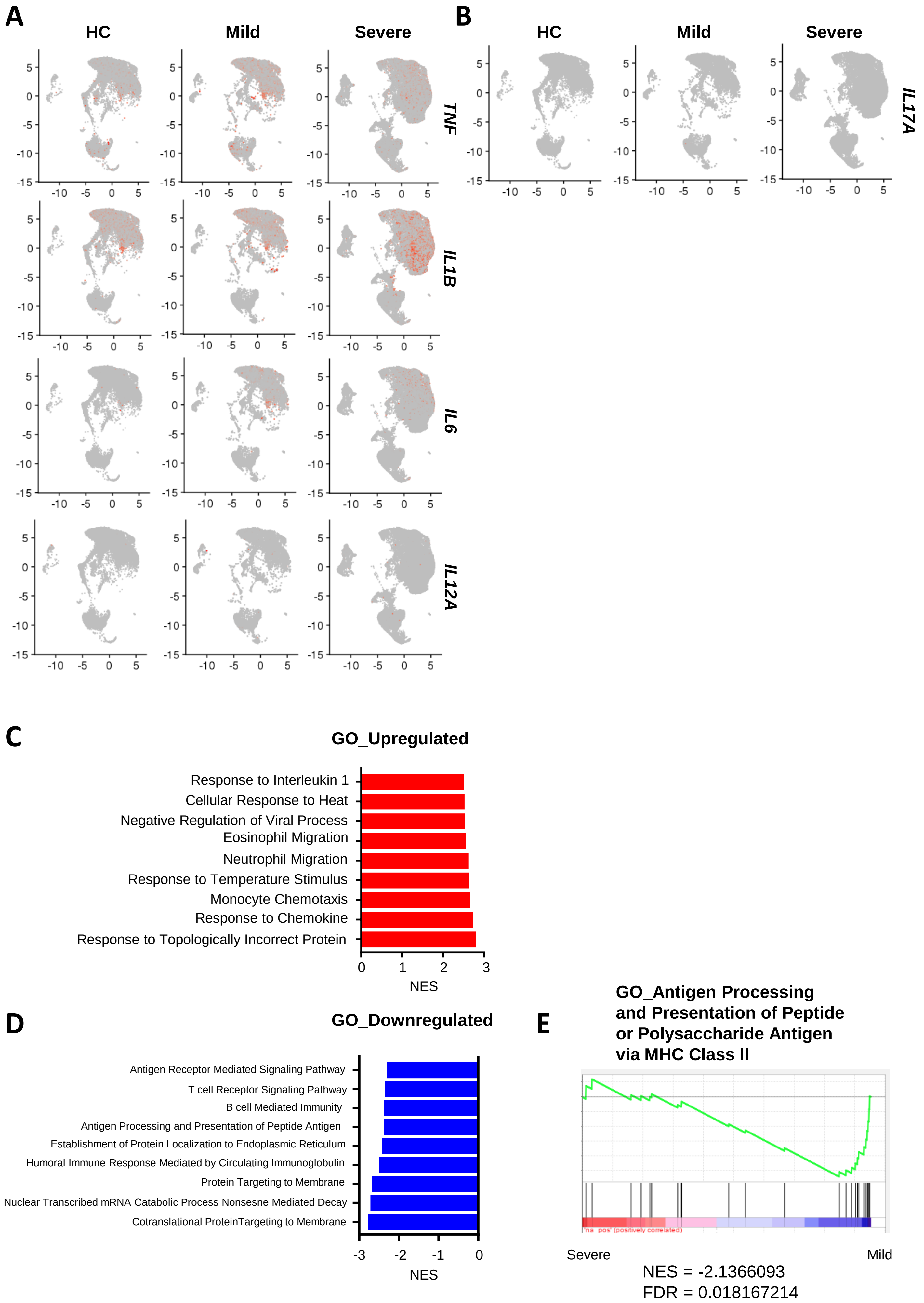


Park et al. Figure S2

**Figure S2. Comparison of characteristics of myeloid cells between severe and mild COVID-19 patients, related to Figure 2. (A)** A feature plot of the expression of proinflammatory cytokines (*TNF, IL1B, IL-6, IL12A*) by groups. **(B)** A feature plot of the expression of *IL17A* by groups. **(C-D)** Top 10 up- **(C)** and downregulated **(D)** pathways in myeloid cells of the severe COVID-19 group compared with the mild group. The p value of all presented pathways was zero. The comparison was dependent on normalized enrichment score (NES) . **(E)** Gene Set Enrichment Analysis of differentially expressed genes between severe and mild patients groups with Gene Ontology (GO) sets for processing and presentation of peptide or polysaccharide antigens using MHC class II.

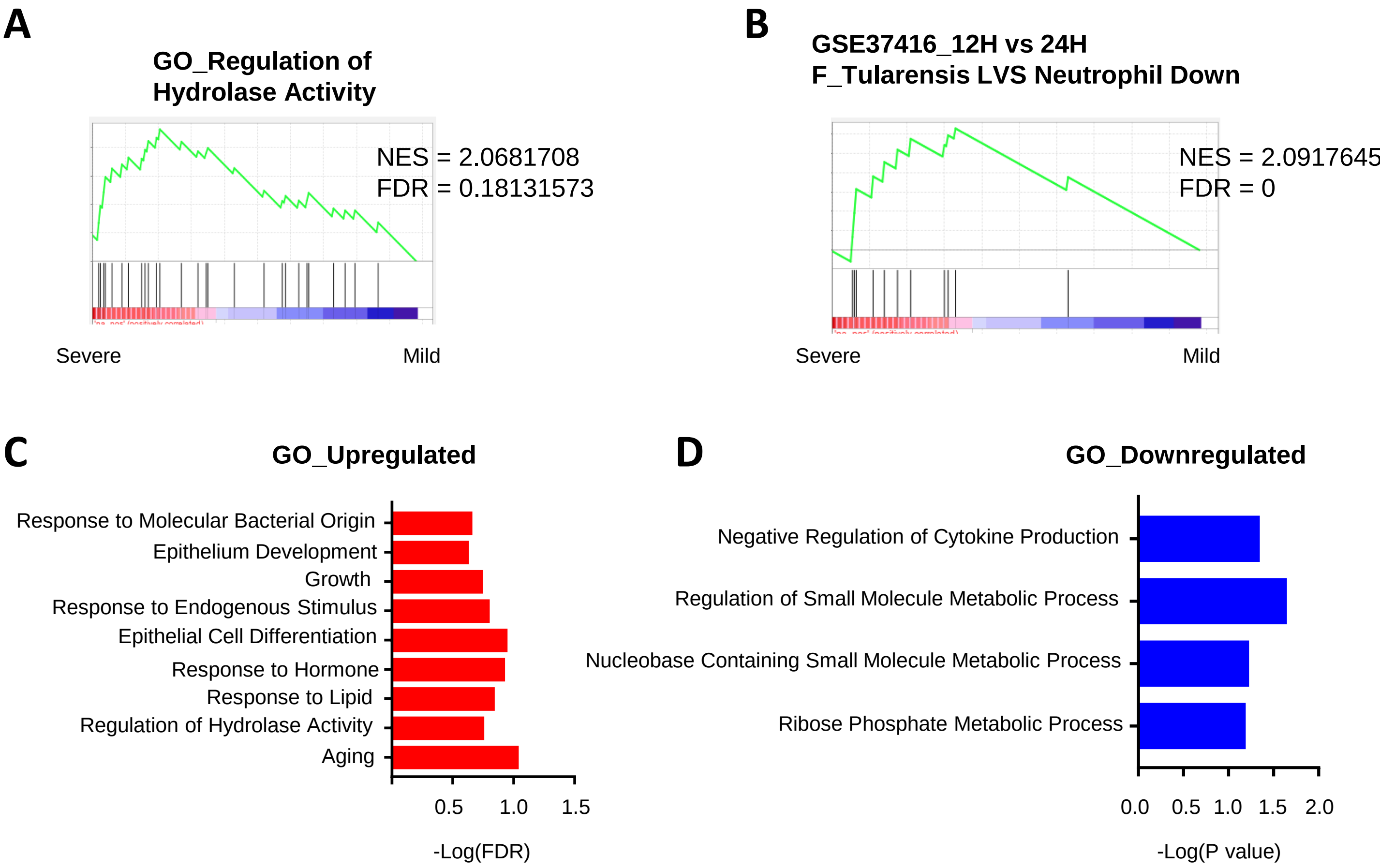


**Figure S3. Characteristics of neutrophils recruited into the lung, related to Figure 3.** **(A-B)** Gene Set Enrichment Analysis of differentially expressed genes between neutrophils from severe and mild COVID-19 patients with gene sets of hydrolase activity **(A)** and downregulated gene sets of neutrophils after 24 h of *F. Tularensis* infection, when compared to 12 h (GSE37416) **(B). (C-D)** Top 10 up- **(C)** and downregulated **(D)** Gene Ontology pathways of neutrophils from severe patients, when compared to mild patients.

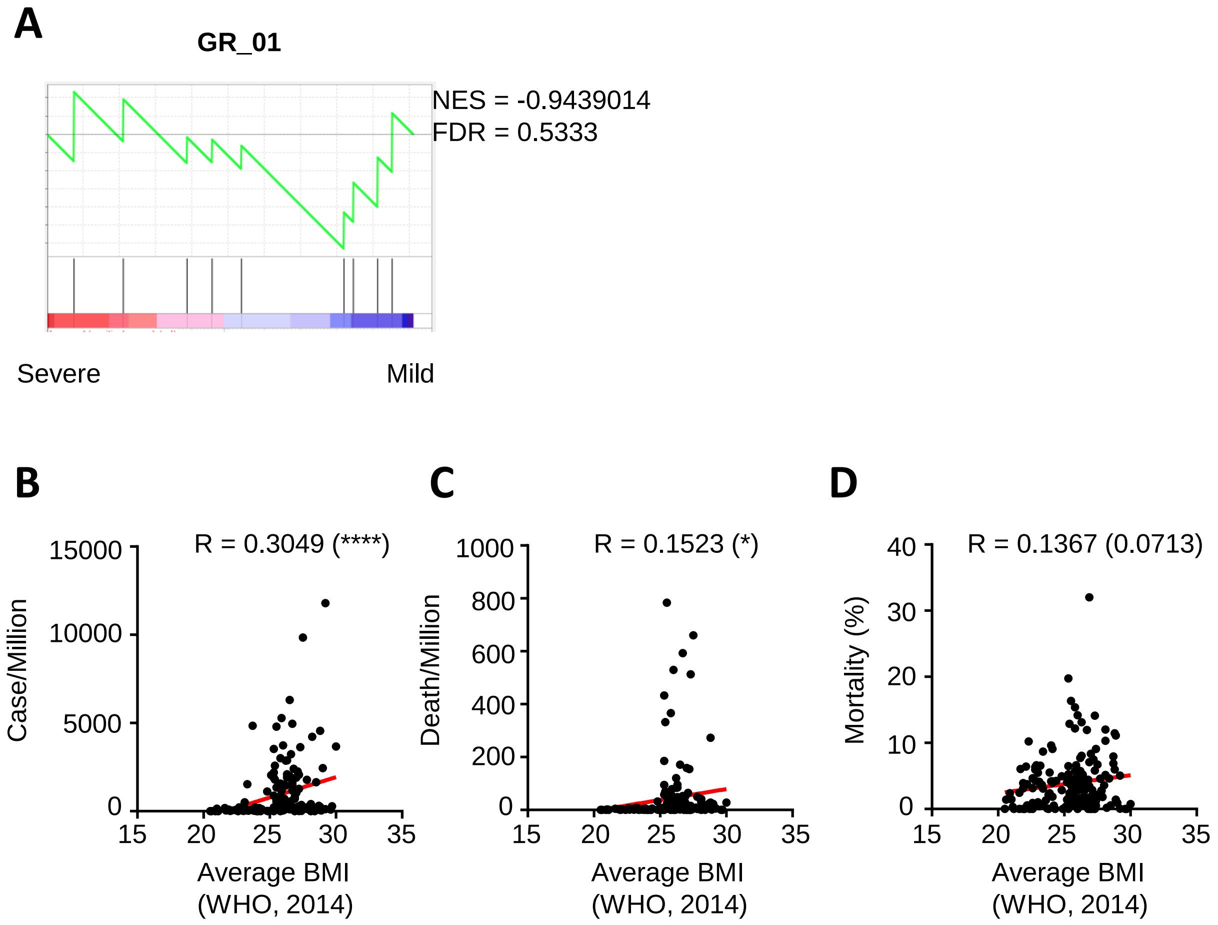


**Figure S4. Relationship between COVID-19 infection and the body mass index (BMI), related to Figure 4. (A)** Gene Set Enrichment Analysis of differentially expressed genes between myeloid cells from severe and mild COVID-19 patients with gene sets about GR_01. **(B)** The correlation between average BMI of countries and the number of detected COVID-19 patients per million people. **(C)** The correlation between the average BMI of countries and the number of deaths by COVID-19 per million people.**(D)** The correlation between the average BMI of countries and mortality (deaths/cases). One-tailed Pearson's test was used for the analyses. *p < 0.05; **p < 0.01; *** p < 0.001; ****p < 0.0001.
